# Association of CYP1A1*2A Polymorphism with Idiopathic Non-Obstructive Azoospermia in A South Indian Cohort

**DOI:** 10.22074/ijfs.2017.4752

**Published:** 2017-09-03

**Authors:** Shalaka S Ramgir, Nishu Sekar, Divya Jindam., Abilash V.G.

**Affiliations:** Department of Biomedical Sciences, School of Bio Sciences and Technology (SBST), VIT University, Vellore, Tamilnadu- 632014, India

**Keywords:** CYP1A1, Restriction Fragment Length Polymorphism, Infertility, Cohort

## Abstract

**Background:**

Infertility is the inability of a couple to conceive after one and a half years of unprotected sex. Male infertility, which accounts for almost half of infertility cases, is considered as a major problem all over the world. The aim of this study was to investigate the association of *CYP1A1* polymorphisms with idiopathic non-obstructive azoospermia in a South Indian cohort.

**Materials and Methods:**

An experimental study was conducted with idiopathic nonobstructive azoospermia. A total of 120 infertile and 80 fertile samples were collected, and DNA was then extracted from all samples. The CYP1A1*2A polymorphism genotyping was carried out by polymerase chain reaction (PCR) and restriction fragment length polymorphism (RFLP).

**Results:**

The genotype distribution of CYP1A1*2A polymorphism showed significant difference between patients and controls. Moreover, the CC genotype was associated with decreased risk of idiopathic non-obstructive azoospermia in comparison with the TT and TC genotypes.

**Conclusion:**

The current experimental study identified that the CT genotype of CYP1A1*2A polymorphism may contribute to the pathogenesis of male infertility in the South Indian population.

## Introduction

Infertility is the inability of a couple to conceive after one and a half years of unprotected sex. It is one of the major medical problems where about 10-15% of couples are affected with infertility of which 50% of these cases are male-related (1). The literature suggests that about 15% of male infertility cases are due to genetics factors (2). Besides genetic and environmental factors, about 30% of cases of infertility in men remain poorly understood in terms of etiology and pathogenesis, and their condition is thus considered idiopathic (3). A decrease in sperm count and motility from 38.18 million/ ml and 61.16% in 1993-1994 to 26.61 million/ ml and 47.14% respectively by 2004-2005 was recorded in a study on the Indian population. Sperm morphology was 40.51% in 1993-1994 and was decreased to 19.75% by 2004-2005 (4). Ageing or environmental toxicants initiate DNA strand break in the spermatozoa of affected males, eventually leading to a mutation in the embryo (5). Genetic factors can be identified in male infertility with congenital hypogonadotropic hypogonadism, congenital absence of vas deferens and primitive testicular failure (6). Epidemiological studies have been unequivocal about the effects of lead (Pb^2+^) and cadmium (Cd^2+^) on hormone concentrations, male fertility and sperm parameters (7).

*CYP1A1* (cytochrome P450, family 1, subfamily A, polypeptide 1) (8) encodes the CYP1A1 enzyme that catalyzes the bioactivation of polycyclic aromatic hydrocarbons (PAHs). In the natural environment, PAHs are capable of forming DNA adducts after it has being activated to generate DNA reactive metabolism. In sperm cells DNA adducts may be considered as a sign of severe DNA damage and infertility is thought to be associated with such damage, which may affect meiotic division during spermatogenesis (9). The four most important enzyme families involved in the metabolism of xenobiotics are the N-acetyltransferase (NAT), cytochrome P450 (P450), glutathione-S-transferase (GST) and microsomal epoxide hydrolase (mEH) enzymes (10). A study on the Chinese population suggested that a CYP1A1 polymorphism may contribute to the pathogenesis of male infertility (11). CYP1A1*2A (T→C substitution at nucleotide 3801 in the 3′-non-coding region; rs464693) is the most prevalent in the Asian population (12). Increase in smoking, alcohol consumption and high exposure to chemicals may lead to infertility. The study was therefore designed to investigate the association of the CYP1A1*2A polymorphism with idiopathic non-obstructive azoospermia and to assess the impact of the status of life style factors on the relationship between the polymorphism and susceptibility to idiopathic non-obstructive azoospermia.

## Materials and Methods

In this experimental study, 120 idiopathic azoospermic men were included but excluding those with known cases such as Y chromosome microdeletion, obstructive azoospermia and Klinefelter syndrome all of which were tested at the Andrology Department, Stanley Medical College and Hospitals, Chennai, India. The age of azoospermic men ranged from 24-38 years and the 80 fertile healthy subjects (control group) in the same age range were included in the study. The criterion for including healthy controls was to have at least one child without assisted reproductive technologies. Couples reported with female factors were excluded from the study. With the help of an experienced urologist at Stanley Hospital, for each patient, a detailed case history was obtained and a clinical examination was carried out. The lifestyle habit and chemical exposure of the probands were recorded including smoking habits, alcohol drinking and exposure to toxic chemicals. Semen was collected from both infertile and fertile males after three days of abstinence from sex and semen volume, sperm count, and motility were recorded. Blood sample from each participant was collected by a physician with written consent. The study was approved by the University Human Ethical Committee of the VIT University.

### Genotype determination

DNA was extracted from 2 ml of venous blood according to lab procedure and stored at +4°C and then subjected to agarose gel electrophoresis. Oligonucleotide sequences of the polymerase chain reaction (PCR) primers were 5ˊ-CAGTGAAGAGGTGTAGCCGC-3ˊ and 5ˊ-TAGGAGTCTTGTCTCATGCC-3ˊ, and the product length was 340 bp. Three μl of DNA was amplified with initial temperature of 95°C for 5 minutes, 30 cycles of denaturation at 94°C for 45 seconds, annealling at 60°C for 50 seconds and extension at 72°C for 1 minute, followed by a final extension at 72°C for 10 minutes in a thermal cycling machine. The 20 μl PCR mixture contained 10 pmol of each forward and reverse primer, 6 μl of master mix, 9 μl of autoclaved miliq water and 4 μl of DNA. The PCR products were separated by gel electrophoresis on a 3% agarose gel containing ethidium bromide (50 μg/μl) and were visualized under UV illumination. The results were analyzed with a gel analysis software (MEDCARE). 2 μl of amplified PCR products were then mixed with 7 μl of nuclease free water, 2 μl of 10X buffer and 2 units of Msp1 restriction enzyme (Eurofins Genomic India pvt Ltd). The digested fragments were visualized on an agarose gel as above. When an Msp1 restriction site was present, the fragment of 340 bp was digested into two fragments of 140 and 200 bp. Homozygotes for the ancestral allele lacked the 140 and 200 bp fragments and had the PCR band of 340 bp while heterozygous individuals had all the three bands and homozygotes for the derived allele has the two smaller bands (9).

### Statistical analysis

Hardy-Weinberg equilibrium deviation was assessed by using the Chi-Square goodness-of-fit test. The difference in genotypic distribution was analyzed using Fisher’s exact test (two-sided). The statistical package used to estimate the odds ratio and 95% confidence intervals was Graphpad Prism 6.1. A P<0.05 was interpreted as statistically significant.

## Results

**Fig.1:**
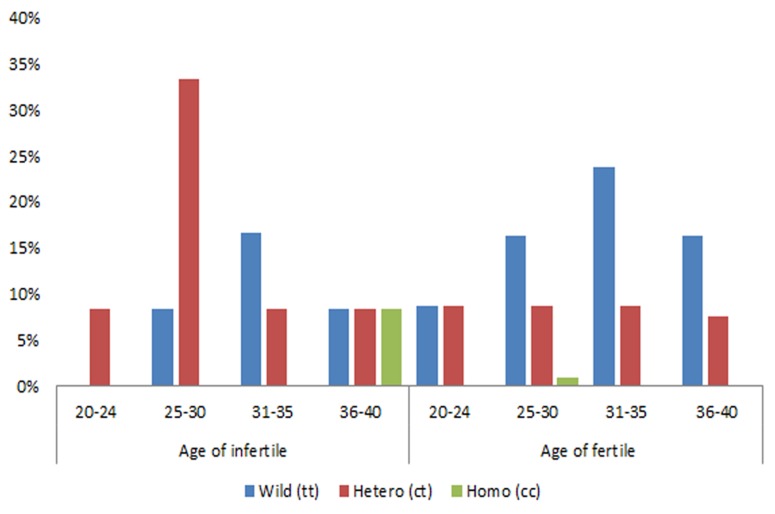
Age-wise distribution of *CYP1A1* polymorphism genotypes in infertile and fertile men.

In total of 120 non-obstructive azoospermic men, higher number of men with CT genotype were observed in the 25-30 age group (>30%) followed TT genotype with (>15%) than any other groups with other genotypes (Fig .1) and this study revealed that 74% of TC, 70% of TT and 40% of CC genotype men had reduced semen volume (<1.5 ml) (Table 1).

**Fig.2:**
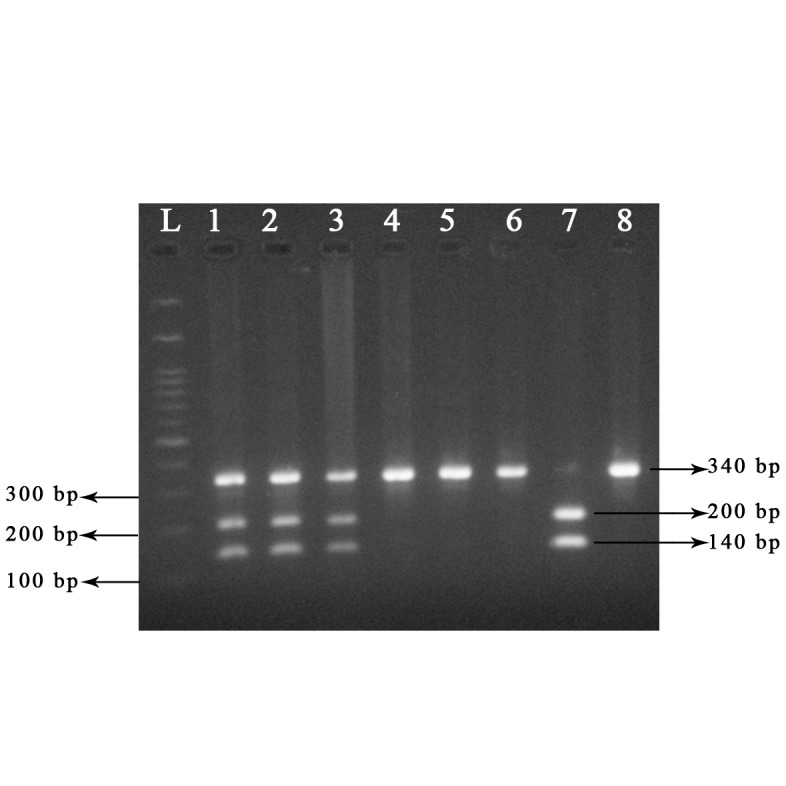
*CYP1A1* gene polymorphism was analyzed by polymerase chain reaction (PCR). Description: Lanes L; Marker, Lanes 1-3; Heterozygous genotype (CT), Lanes 4-6 and 8; Homozygous wild (TT), and Lane 7; Homozygous mutant (CC).

**Fig.3:**
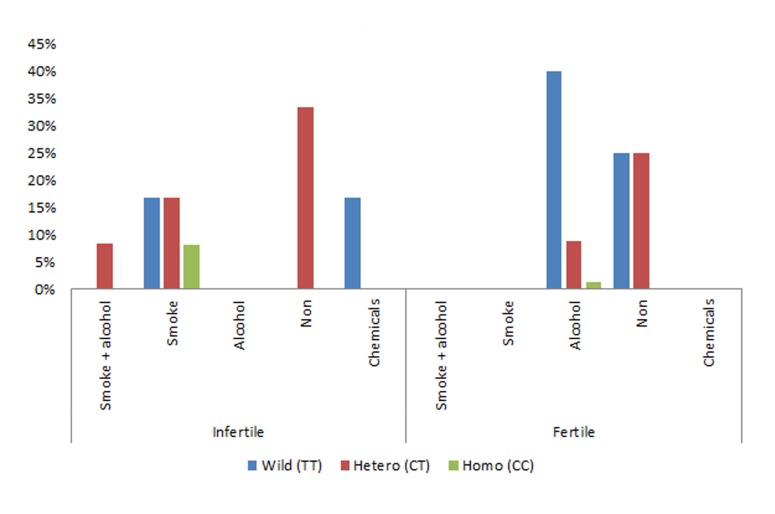
Association of smoke, alcohol and chemical exposure with the *CYP1A1* polymorphism in infertile and fertile men.

Bands corresponding to the 340 bp PCR fragment were observed, confirming amplification of this region of *CYP1A1*. The RFLP analysis of CYP1A1*2A polymorphism results of the 120 patients (Fig .2, RFLP results of a few samples), showed that the genotype counts were 70 heterozygous, 40 homozygous for the ancestral allele and 10 homozygous for the derived allele. In the control group the counts were 27, 53 and 1 respectively. The observed frequency of patients with homozygous CC was 8.34% of which all were exposed to smoking, the percentage of homozygous TT was 33.34% of which 50% were exposed to smoking and 50% were exposed to chemicals, and the percentage of heterozygous CT was 58.34% of which 57.14% were not exposed to any harmful chemicals and 28.57% were exposed to smoking and 14.2% were exposed to both alcohol as well as smoking. In the control group homozygous CC was 1.25% of which 100% were exposed to smoking, the percentage of homozygous TT was 65.00% of which 61.53% were exposed to alcohol and 38.46% were not exposed to any harmful chemicals, and the percentage of heterozygous CT was 33.75% of which 25.92% were not exposed to alcohol and 74.07% were not exposed to any harmful chemicals (Fig .3).

The differences in allele frequencies of this CYP1A1*2A polymorphism between fertile and infertile men were found to be statistically significant (P=0.0001). Differences in genotypic was also observed between infertile and fertile men (P=0.0001). The semen analysis report showed the frequencies of TT, CT and CC genotypes in azoospermic men were found to be 23.33, 43.33 and 3.33% in patients with less than 1.5 ml of reduced semen volume and 10, 15, 5% in patients with more than 1.5 ml of reduced semen volume respectively. In fertile controls with normal semen volume, we observed 65% TT, 33.75% CT and 1.25% CC genotypes (Table 2).

**Table 1:** Semen volume in relation to the *CYP1A1* polymorphism in azoospermic and fertile men


	Group and genotype	Semen volume		
		Reduced (<1.5 ml)	Normal (>1.5 ml)

Infertile	TT (wild) n=40	28	12
	TC (hetero) n=70	52	18
	CC (homo) n=10	4	6
Fertile	TT (wild) n=52	0	52
	TC (hetero) n=27	0	27
	CC (homo) n=1	0	1


**Table 2:** Genotype frequencies of the CYP1A1*2A polymorphism among infertile and fertile men (controls) and their association with male infertility


CYP1A1 genotypes	Fertile men (Control) n=80	Infertile men (Patients) n=120	P value	OR (95% CI)

TT (Wild)	52	40	Reference	
TC (Hetero)	27	70	0.0001^*^	3.43 (1.87-6.29)
CC (Mutant)	1	10	0.0001^*^	13 (1.597-105.8)
TC+CC	28	80	0.0001^*^	3.714 (2.046-6.741)


OR; Odds ratio and CI; Confidence interval.

## Discussion

CYP1A1 is an important phase I enzyme and plays a key role in the metabolism of lipophilic xenobiotics. The enzyme is vitally expressed in male reproductive organs and its polymorphisms may be a determinant of individual susceptibility to infertility. Metabolic activation or inactivation of xenobiotics is catalyzed by hemethilate enzymes like CYP1A1, which catalyzes PAHs in the first step of metabolism. For instance, the process of converting the carcinogen benzo[a]pyrene (B[a] P) to its ultimate DNA-binding form is metabolized by CYP1A1 (13). These metabolites have been shown to cause small cell lung carcinoma (14), recurrent pregnancy loss (15), coronary artery disease and diabetes (16). It is thought that CYP1A1 also plays a vital role in metabolism of endogenous substrates like steroid hormones through catalyzing the hydroxylation of 17b-estradiol at the C-2 position (17-19).

The genotypic distribution of CYP1A1*2A polymorphism in the infertile male group deviated from the Hardy-Weinberg equilibrium. There have not been any reports describing such an incompatibility for *CYP1A1* polymorphisms in the South Indian population. To remain in the Hardy-Weinberg equilibrium, the population must be very large and must follow random mating. Our study population is relatively small and consanguineous marriages are 5% common in this population. The observed incompatibility may thus be inherent to the studied population. In the overall analysis, we found that individuals heterozygous for this polymorphism had an increased risk. In the subsequent analysis, we found that patients exposed to smoking, alcohol or chemicals have an overrepresentation of the homozygous ancestral genotype TT, leading to male infertility. The patients with smoking, alcohol consumption and high exposure to chemicals may also have an increased risk in heterozygous type polymorphism leading to male infertility.

It is suggested that in infertility, genetic polymorphisms of xenobiotic metabolism may play an important role (20). Based on an Indian study, the pathogenesis of male infertility was associated with the CC genotype of the CYP1A1*2A polymorphism (9). Besides the study on the Indian population, other studies have shown that being homozygous for the CYP1A1*2A variant increased susceptibility to estrogen-related breast cancer in African-Americans (21). However, a case-control study on Japanese women showed a decreased risk with homozygous CYP1A1*2A among breast cancer patients (22). A study of *CYP1A1* in the Chinese population showed that variants in this gene may contribute to the pathogenesis of male infertility in the Han population (10). To completely understand the etiology of idiopathic male infertility, an understanding of the complex gene-environment interactions is necessary. This is particularly relevant for genes such as CYP1A1 which is in direct contact with environmental toxins. Smoking, which was reported at a moderately high percentage in the infertile group of this study, could be an additional contributory factor in the development of male infertility by increasing levels of PAH in the body (23). The study carried out by Abilash et al. (24) estimated the frequency of Y chromosome microdeletion in infertile men to explore the effect of smoking, alcohol drinking, chemical exposure and cellular chromosomal aberration among 34 azoospermia and 55 oligospermia patients. They found that the chromosome aberrations per cell in azoospermia and oligospermia were higher than that of the control. The percentage of microdeletion observed in unexposed azoospermia had 15%, azoospermia smokers 22%, azoospermia smokers and alcoholics 25%; whereas the unexposed oligospermia had 7%, oligospermia smokers had 12%, and oligospermia smokers and alcoholics had 37%. Based on these results, they concluded that the etiology of male infertility may differ between ethnicities and smoking, alcohol drinking and chemical exposure may have deleterious effects on human fertility (24).

## Conclusion

Our study indicates that the CT genotype of CYP1A1*2A may contribute to the pathogenesis of idiopathic non obstructive azoospermia. This result thus suggests that the relationship between this genetic variation and the vulnerability to the disease depends on personal habits such as smoking, alcohol drinking and other environmental factors such as exposure to chemicals and heavy metals. Since this study is a preliminary step in investigating this association, further studies are needed to identiyfing the underlying mechanism and to validate our results.
